# Supplementary figures and images for: FastProject: a tool for low-dimensional analysis of single-cell RNA-Seq data
Source: BMC Bioinformatics. 2016 Aug 23;17(1):315. doi: 10.1186/s12859-016-1176-5 (PMC4995760; doi:10.1186/s12859-016-1176-5)

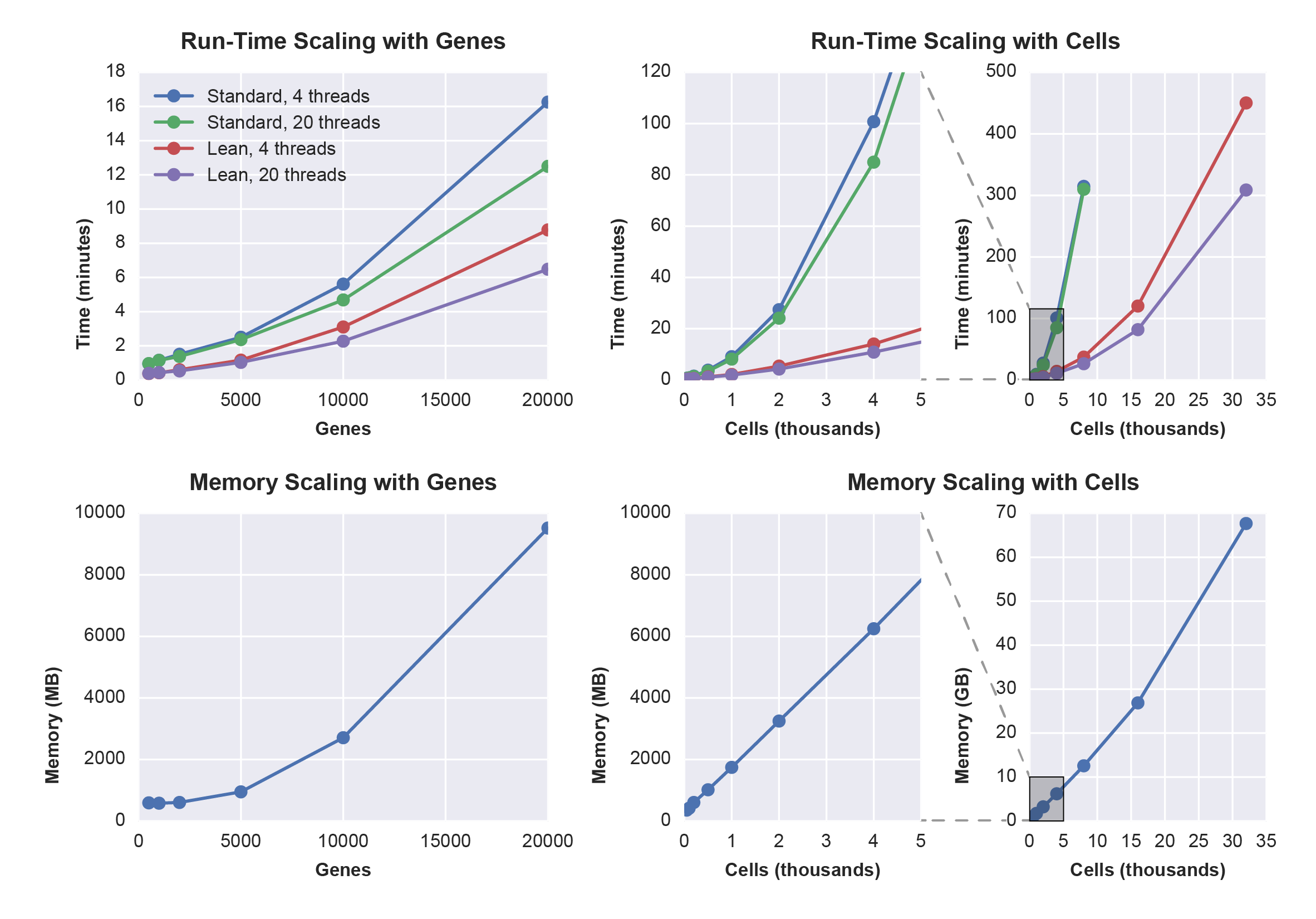

Supplement: Additional file 1 — Benchmarking FastProject. Memory and run-time are measured as the number of genes and cells vary. When varying the number of genes, 200 cells were used, and when varying the number of cells, an input matrix of 2000 genes was used. “Lean” mode is an option that may be selected at run-time which disables some projection methods that were found to scale poorly with the number of samples (MDS, Spectral Embedding) as well as removing extra filter steps (HDT, Fano Factor). Trials run using a compute cluster with 40 Intel Xeon E5-2690 processors at 3 GHz, but intentionally limited to either 4 or 20 threads during the run-time tests. (PNG 188 kb) [file 12859_2016_1176_MOESM1_ESM.png]

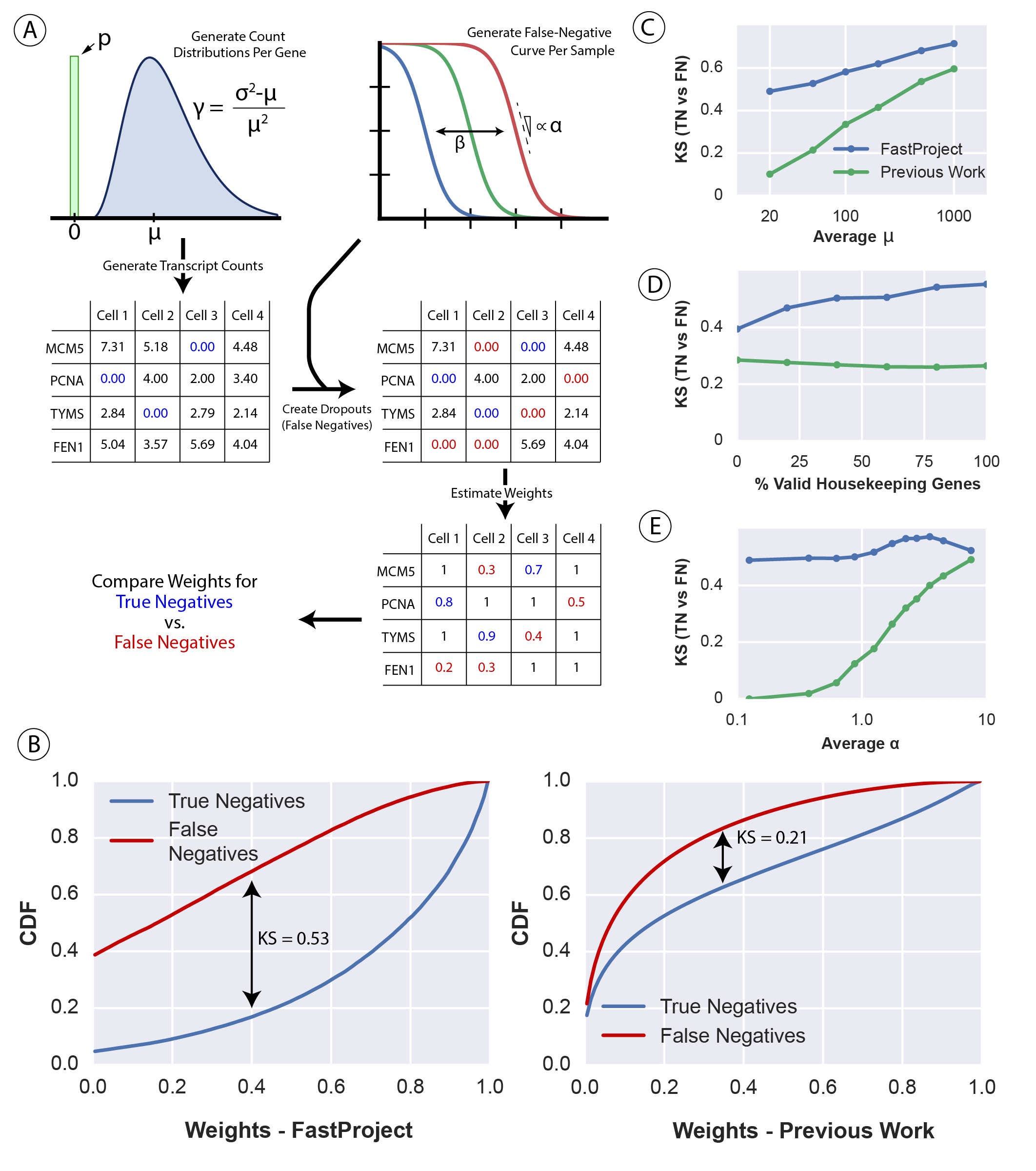

Supplement: Additional file 2 — Simulation of False-Negative Weight Estimation Procedure. A) A simulation to test FastProject’s ability to distinguish genes that are biologically inactive (True Negatives) from genes dropped out due to technical artifacts (False Negatives). B) The Kolmogorov-Smirnov (KS) statistic is used to distinguish between distributions of true and false negatives. Here it can be seen that the true negatives tend to be assigned higher weights. This is contrasted with the weighting scheme used in a previous study (Gaublomme et al. 2015 [7]) in which the true and false negatives are not as differentiated. C-E) The KS statistic’s for either scheme, as in (B), as simulation parameters are varied. C) Tests varying the parameter which controls the mean of the exponential distribution from which μ is drawn. D) False-negative curves are estimated using a mixture of housekeeping genes and non-housekeeping genes. It can be seen that the choice of good housekeeping genes is beneficial, but not critical. E) The steepness of the generated false-negative curves, α, is varied. (PNG 258 kb) [file 12859_2016_1176_MOESM2_ESM.png]

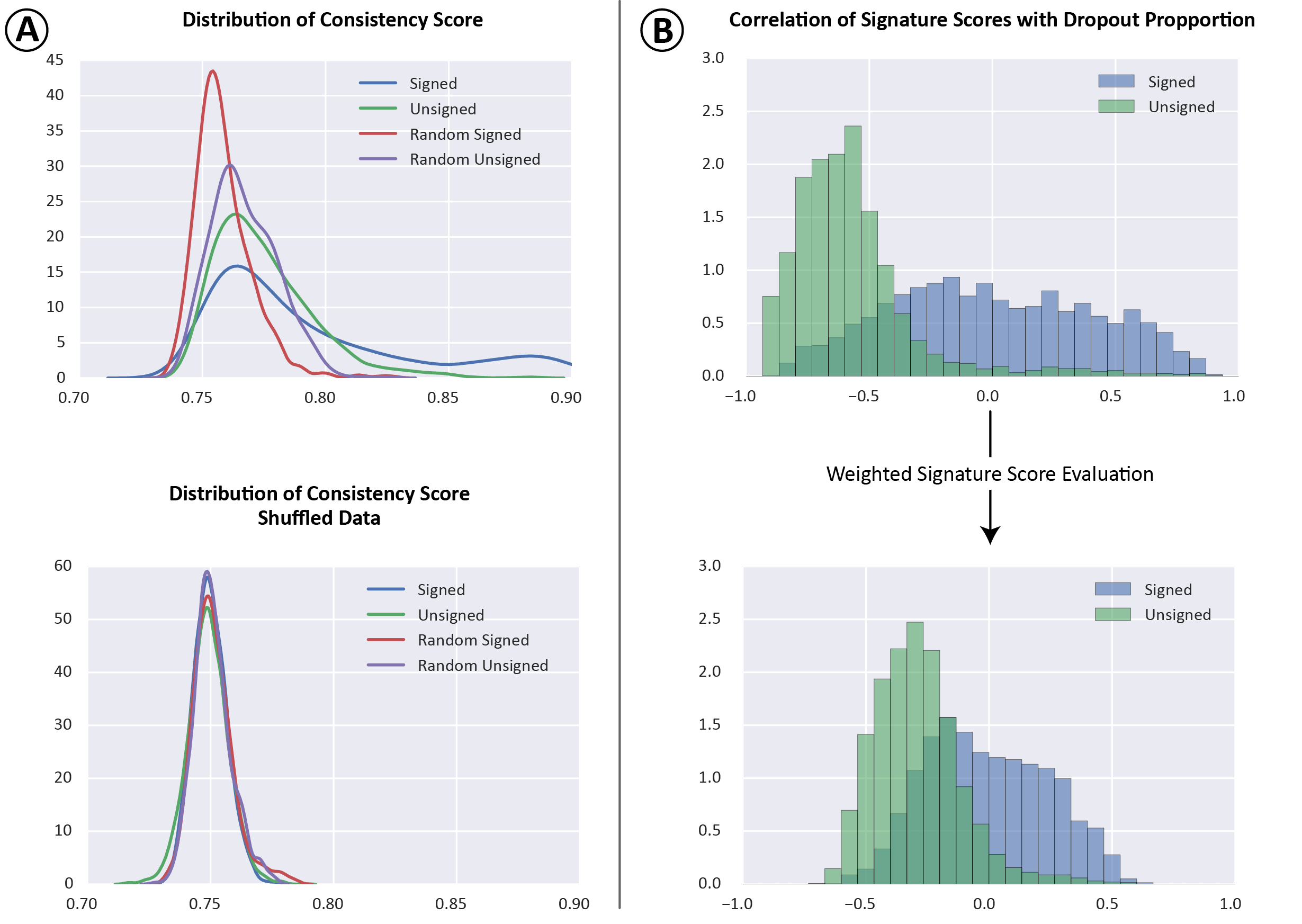

Supplement: Additional file 3 — Behavior of Signature Scores: Alternate Data Set. Behavior of signature scores calculated from the LPS-stimulated dendritic cells of Shalek et al. 2014 [1]. A) Distribution of Signature/Projection consistency scores across four different types of signatures, Signed (signed immunological signatures from MSigDB), Unsigned (various unsigned hallmark and pathway signatures from MSigDB), Random Signed (signed signatures with randomly selected genes), and Random Unsigned (unsigned signatures with randomly selected genes). Lower panel shows distributions from the same signatures, run on data in which gene expression levels have been shuffled within each cell. B) Distribution of the Pearson’s correlation coefficient between signature scores and a confounding variable - the proportion of undetected genes in a sample. Upper plot shows correlations when signature are calculated by simply taking the unweighted average of log expression level for genes in the signature. Lower panel shows the effect of using the weighted method presented here. (PNG 152 kb) [file 12859_2016_1176_MOESM3_ESM.png]

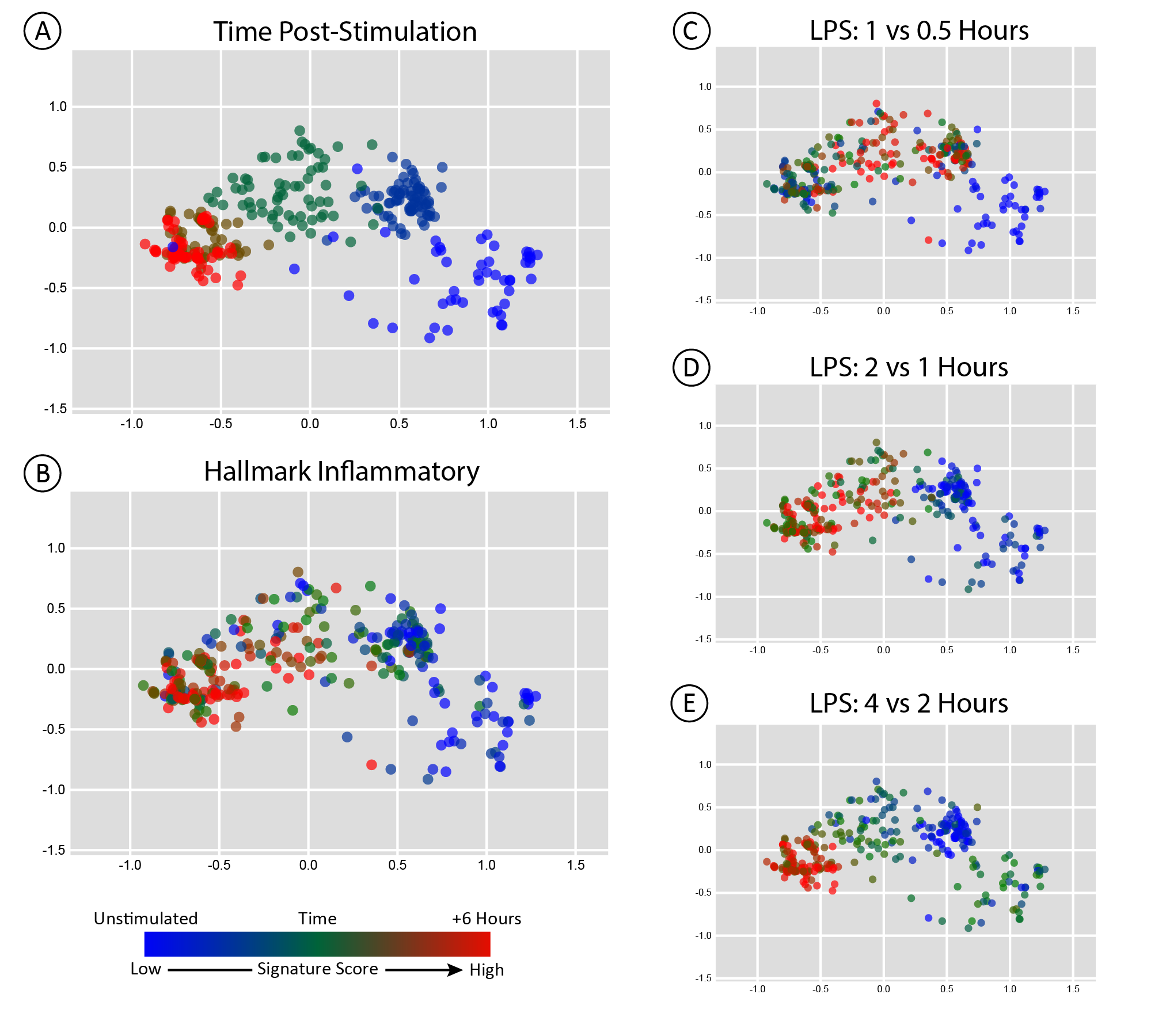

Supplement: Additional file 4 — Signature-Projection Pairings. An example of signature/projection pairings from FastProject run on a subset of cells from Shalek et al. 2014 [1]. Analysis was run on bone marrow dendritic cells, stimulated with LPS and sequenced after 1–6 h. For comparison purposes, the same projection (ISOMAP on genes selected by the Fano Factor filter) is used for each plot. A) The arrangement of cells in the ISOMAP projection can be seen to largely agree with the time of sequencing, post stimulation. B) Cells at later times score higher on the HALLMARK_INFLAMMATORY signature from the MSIGDB collection. C-E) Temporal signatures generated from Amit et al. 2009 [36]. Signatures generated by taking the top 200 up and down-regulated genes, between adjacent time points, and removing genes with less than a 2-fold difference. Changes in these signature scores can be seen to largely correspond with the temporal labels in (A). All signature/projection pairings, A-E, were found to be significant by FastProject (p≤0.05). (PNG 339 kb) [file 12859_2016_1176_MOESM4_ESM.png]
